# Clinical pathological characteristics of breast cancer patients with secondary diabetes after systemic therapy: a retrospective multicenter study

**DOI:** 10.1007/s13277-015-3380-8

**Published:** 2015-04-09

**Authors:** Li Juanjuan, Wei Wen, Liu Zhongfen, Chen Chuang, Cheng Jing, Gong Yiping, Wang Changhua, Yu Dehua, Sun Shengrong

**Affiliations:** 1 Department of Breast and Thyroid Surgery, Wuhan University, Renmin Hospital, Wuhan, 430060 China; 2 Cancer Center, Union Hospital, Tongji Medical College, Huazhong University of Science and Technology, Wuhan, 430022 China; 3 Department of Breast Surgery, Hubei Cancer University, Wuhan, 430079 China; 4 Department of Pathology and Pathophysiology, Wuhan University School of Basic Medical Sciences, Wuhan, 430071 China

**Keywords:** Breast cancer, Type 2 diabetes, Systemic therapy, Clinical features, Pathological features

## Abstract

The objective of this study was to investigate the clinical pathological characteristics of breast cancer (BC) patients with secondary diabetes after systemic therapy without preexisting diabetes. A total of 1434 BC patients received systemic therapy and were analyzed retrospectively. Fasting plasma glucose (FPG) levels were monitored prior to the treatments, during the course of systemic therapy, and at the follow-up visits. Cox regression models were used to estimate the associations between the clinical pathological characteristics of BC and the cause-specific hazard of developing secondary diabetes. Among the 1434 BC patients, 151 had preexisting type 2 diabetes. Of the remaining 1283 patients with normal FPG levels prior to the systemic therapy, 59 developed secondary diabetes and 72 displayed secondary impaired fasting glucose (IFG) over a mean follow-up of 41 months. The prevalence of secondary type 2 diabetes in BC patients was 4.6 % (59/1283), which was obviously higher than that of the normal control group (1.4 %, *P* < 0.001). The percentage of older patients (*P* < 0.05), menopausal patients (*P* < 0.001), and obese patients (*P* < 0.01) tended to be lower in the secondary diabetic group. In addition, these patients with secondary diabetes had later pathological stages (*P* < 0.01), more lymph node metastasis (*P* < 0.05), negative estrogen receptor (ER) expression (*P* < 0.05), and smaller size of tumors (*P* < 0.05). After adjusting for age and BMI, the risk of developing secondary diabetes and IFG in subjects with later pathological stage BC (hazard ratio (HR) = 1.623; 95 % confidence interval (CI) 1.128–2.335 (*P* < 0.01)), negative progesterone receptor (PR) expression (HR = 0.530; 95 % CI 0.372–0.755 (*P* < 0.001)), positive human epidermal growth factor receptor 2 (HER2) expression (HR = 1.822; 95 % CI 1.230–2.700 (*P* < 0.01)), and more lymph node metastasis (HR = 1.595; 95 % CI 1.128–2.258 (*P* < 0.01)) was significantly higher. In conclusion, this study shows that an increase in the incidence of diabetes among breast cancer survivors after systemic therapy, especially the patients with later pathological stages, more lymph node metastasis, negative hormone receptor expression, and positive HER2 expression. Our study suggests that greater diabetes screening and prevention strategies among breast cancer patients after systemic treatment are needed in China.

## Introduction

Breast cancer (BC) and diabetes mellitus are common diseases and major health care problems among females in the world. The overall prevalence of diagnosed diabetes was 11.6 % among the Chinese adults, 12.1 % among men, and 11.0 % among women [1]. Breast cancer accounted for 25.2 % of all cancers and 14.7 % of cancer deaths in females worldwide in 2012 [2]. In China, breast cancer is currently the most common malignant tumor, and as a result, it is the second leading cause of cancer deaths in women [3].

Many BC patients are also diagnosed with preexisting diabetes, possibly due to the shared etiology and signaling pathways [4, 5]. These include old age; obesity; genetic predisposition; a sedentary lifestyle; metabolic syndrome; as well as biological factors such as hyperinsulinemia, hyperglycemia, and chronic inflammation [6–10]. Epidemiological studies suggest that type 2 diabetes mellitus (T2DM) increases breast cancer risk and thereafter goes with an increased mortality. A study by Larsson et al. suggested that females with diabetes had a 20 % increased risk of postmenopausal breast cancer. Interestingly, the statistical association between diabetes and breast cancer is significantly stronger in Asia than that in North America [4]. Diabetes may influence therapy choices, affect therapeutic toxicities, and cause worse outcomes. Further studies suggested that diabetes was associated with a 40–50 % increased mortality in BC patients [7, 8]. In addition, the risk of developing BC increased significantly after the long-term use of insulin glargine [11].

On the other hand, emerging evidence suggests that females with BC have an increased risk of developing diabetes. Lipscombe et al. reported that 9.7 % of older females with early-stage BC developed diabetes after adjuvant therapy for a median follow-up of 5.8 years. The risk started to increase 2 years after diagnosis, especially for the patients who received adjuvant chemotherapy [12]. A recent population-based retrospective study found that previously undiagnosed diabetes was associated with advanced-stage cancer and increased mortality [13]. However, no study has been reported that whether secondary diabetes is associated with chemotherapy in both young and older females with breast cancer and how it is affected by clinical pathological characteristics.

In this study, we focused on the clinical pathological characteristics of breast cancer patients with secondary diabetes after systemic therapy without preexisting diabetes and tried to provide the clinical guidance for these patients.

## Subjects and methods

### Subjects

With Ethics Committee approval, we retrospectively evaluated 1434 patients with BC aged 20–80 years who were treated in Renmin Hospital of Wuhan University, Hubei Cancer Hospital, and Union Hospital of Tongji Medical College, Wuhan, China, between January 2008 and July 2014. All the patients were diagnosed by pathological examination. The diagnosis of type 2 diabetes mellitus was based on the following criteria: the medical history and prescription records which were obtained by doctors during interview and the definition of the 1999 World Health Organization (WHO), including a fasting plasma glucose (FPG) ≥7.0 mmol/L, a random plasma glucose ≥11.1 mmol/L, or a 2-h plasma glucose ≥11.1 mmol/L during an oral glucose tolerance test (OGTT). Impaired fasting glucose (IFG) was defined as values above normal, but below the diagnostic cutoff for diabetes (≥6.1 to <7.0 mmol/L) [14]. All the patients accepted the following exclusion criteria: (1) type 1 diabetes mellitus, (2) history of cancer, and (3) male patient. Finally, all the patients included in our study had complete data available and received systemic treatment including surgery and chemotherapy.

### Data collection

Both demographic information and medical records were collected for analysis. The demographic information included age at the time of breast cancer diagnosis, height, and weight. The medical history recorded included a previous history of diabetes, menopausal status, age of onset of menarche, family history of breast cancer, stage of breast cancer at the time of diagnosis, hormonal receptor status (estrogen, progesterone) by immunohistochemistry (IHC) assay, human epidermal growth factor receptor 2 (HER2)/neu status by either an IHC assay or a fluorescence in situ hybridization (FISH) assay (HER2-positive means FISH-amplified or IHC3+; HER2-negative means FISH-non-amplified or IHC 0–1+; IHC2+ is defined as borderline which requires FISH assay.), lymph node status, surgical therapy, chemotherapeutic records, laboratory results, recurrence of breast cancer, history of bilateral breast cancer, and survival of the patients since the diagnosis of cancer (in months). Death was validated using social security records. Obesity (BMI ≥25 kg/m^2^) was diagnosed according to the Asia-Pacific criteria [15]. Detailed information is presented in Table 1.

**Table 1:** General characteristics of patients with diabetes and impaired fasting glucose

General characteristics	Preexisting diabetes (*n* = 151)	Secondary diabetes (*n* = 59)	Secondary IFG (*n* = 72)	*P* values
Age (years), mean ± SD	54.43 ± 8.97	54.90 ± 9.35	51.07 ± 9.62	0.021
Urban/rural status				
Urban	98 (64.90 %)	44 (74.58 %)	48 (66.67 %)	0.401
Rural	53 (35.10 %)	15 (25.42 %)	24 (33.33 %)	
Family history of BC in first degree relatives				
Positive	7 (4.64 %)	1 (1.70 %)	1 (1.39 %)	0.332
Negative	144 (95.36 %)	58 (98.30 %)	71 (98.61 %)	
Age of onset of menarche (years), mean ± SD	13.82 ± 1.45	13.08 ± 1.32	12.73 ± 1.33	<0.001
Menopause status				
Premenopausal	49 (32.45 %)	17 (28.81 %)	35 (48.61 %)	<0.001
Postmenopausal	102 (67.55 %)	42 (71.19 %)	37 (51.39 %)	
Age at first live birth (years)				
20–29	140 (92.72 %)	53 (89.83 %)	65 (90.28 %)	0.728
≥30	11 (7.28 %)	6 (10.17 %)	7 (9.72 %)	
Breastfeeding (months)				
0–6	15 (9.93 %)	6 (10.17 %)	9 (12.50 %)	
≥7	136 (90.07 %)	53 (89.83 %)	63 (87.50 %)	0.837
BMI (kg/m^2^), mean ± SD	24.86 ± 2.44	23.92 ± 1.59	23.19 ± 1.31	<0.01

Data are expressed as means ± SD or *n* (%)

Previously diagnosed diabetes among the breast cancer patients was determined on the self-report and the medical record. As part of routine clinical care, FPG levels were determined presurgery, before the commencement of each chemotherapy cycle, and at 3–6 months intervals after the final cycle. The management of diabetes (including dietary control, oral drugs, or insulin) and the discontinuation of chemotherapy were recorded. All the patients that were diagnosed with diabetes were referred to an endocrinologist. Cohort entry date was defined as the date of breast cancer diagnosis.

### Treatment

All BC patients received surgical treatment and chemotherapy. In addition, most patients with diabetes were treated with oral hypoglycemic agents or insulin. The systemic treatment for BC was performed according to guidelines and standard protocols. All BC patients received modified radical or simplified mastectomy. Chemotherapy included anthracycline-based combined chemotherapy, paclitaxel/docetaxel alone or combined with other drugs, and second-line chemotherapy regimen (such as capecitabine, gemcitabine, platinums, and vinorelbine). Adverse events (AE) were documented daily during chemotherapy and were classified according to the Common Terminology Criteria for Adverse Events (version 4.0) for final analysis [16]. Radiotherapy, endocrine therapy, and HER2-targeted therapies were given following guidelines and standard protocols.

### Outcome and follow-up

Our primary outcome was a diagnosis of diabetes from cohort entry date until July 2014. Diabetes was identified according to the definition of the 1999 WHO [14]. Persons in the cohort were followed until the incidence of diabetes, the recurrence of breast cancer, the occurrence of a new malignancy, death, or the end of the follow-up period.

### Statistical analysis

Data were stored in an Excel database by three research assistants, and descriptive statistics were calculated for all the study variables. The basic conditions and clinical pathological characteristics of the patients were tested using the *Χ* ^2^ test. The median onset time of secondary diabetes was analyzed between each group using a Kaplan–Meier analysis, and the significance test was performed using the log-rank test. The Cox proportional hazard model was used to examine the correlation between each clinical pathological characteristics and the occurrence of secondary diabetes. The factors included in the Cox multifactorial regression model were both statistical and clinical factors, including age, menopausal status (post- and premenopausal), pathology reports of the lymph nodes (with and without lymph node metastasis), TNM stage (early and advanced stage), expression of estrogen receptor (ER; positive and negative), expression of progesterone receptor (PR; positive and negative), and expression of HER2 (positive, negative, and borderline). Statistical analysis was performed using SPSS (version 17.0). All tests of statistical significance were based on a two-sided *P* < 0.05.

## Results

### Increased incidence of BC patients with secondary diabetes following systemic therapy

**Fig. 1:**
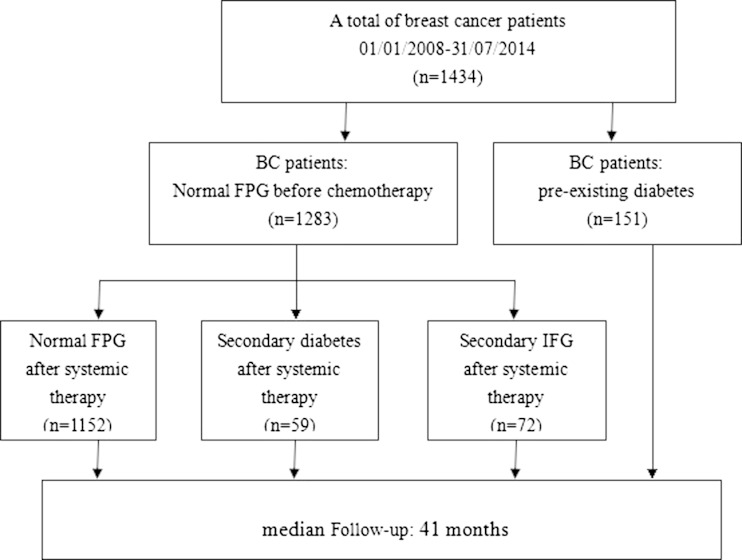
Patient flow diagram

The median follow-up time for this study was 41 months (16–66 months). A total of 1434 BC patients treated with systematic therapy with available FPG data were included in the study. Of these, 10.5 % (151/1434) had preexisting diabetes. The FPG was measured with the remaining 1283 patients without preexisting diabetes during each chemotherapy cycle and at 3–6 months intervals after the final cycle, based on the available data and follow-up results. There were 89.8 % (1152/1283) of the patients with normal FPG levels throughout the whole study period. Approximately 10.2 % (131/1283) of the patients developed hyperglycemia during systemic therapy, including 5.6 % (72/1283) with secondary IFG and 4.6 % (59/1283) with secondary diabetes. Detailed information describing these three groups is summarized in Fig. 1.

In addition, 1361 females without BC or preexisting type 2 diabetes who received an annual routine physical examination at Renmin Hospital of Wuhan University were followed up for 3 year as the normal control group. Of these, 3 % (41/1361) developed hyperglycemia, including 1.6 % (22/1361) with IFG and 1.4 % (19/1361) with diabetes. These percentages were significantly lower than those of BC patients (*P* < 0.001).

### Clinical pathological characteristics of BC patients with secondary diabetes and IFG

The clinical pathological characteristics of the secondary diabetic group and control groups are summarized in Table 1. The mean age of patients with preexisting diabetes was 54.43 ± 8.97 years, which was older than that of the secondary IFG BC group (51.07 ± 9.62 years, *P* < 0.05). The mean age of patients with normal FPG during the follow-up was 43.33 ± 3.59 years, which was younger than that of the other groups (*P* < 0.001). Among the BC patients with preexisting diabetes, the percentage of postmenopausal patients was 67.55 %, which was significantly higher than that of the groups with secondary IFG and diabetes (*P* < 0.001), while the percentage of postmenopausal patients was 42.62 % among the BC patients with normal FPG during the follow-up, which was significantly lower than the other groups (*P* < 0.001). The mean age of onset of menarche in the secondary IFG BC patients was significantly younger than that of the other two groups (*P* < 0.001). BMI of patients with preexisting diabetes was significantly higher than that of the other groups (*P* < 0.01). There were no significant differences in urban/rural habitation, family history of BC in first degree relatives, and age at first live birth and breastfeeding.

Of the patients who developed secondary diabetes, 52.5 % (31/59) were diagnosed initially with advanced-stage BC (stages III/IV), while 31.1 % (47/151) of patients with preexisting diabetes were diagnosed initially with advanced BC. The results show that the percentage of advanced-stage BC was higher in the secondary diabetes group (Table 2, *P* < 0.01). In the secondary diabetes group, patients tend to have more lymph node metastasis than the other two groups (*P* < 0.05). In addition, patients with preexisting diabetes had larger average tumor sizes than the patients with secondary diabetes and secondary IFG (*P* < 0.05). Meanwhile, in the secondary diabetes group, patients tended to have higher percentages of negative ER expression than the other two groups (*P* < 0.05). There were no significant differences in other indicators among these groups (Table 2).

**Table 2:** Clinical pathological characteristics and follow-up of breast cancer patients with secondary diabetes or impaired fasting glucose (IFG)

Pathological characteristics	Preexisting hyperglycemia (*n* = 151)	Secondary diabetes (*n* = 59)	Secondary IFG (*n* = 72)	*P* values
Stage				0.038
I	35 (23.18 %)	10 (16.95 %)	16 (22.22 %)	
II	69 (45.70 %)	18 (30.51 %)	35 (48.61 %)	
III	45 (29.80 %)	31 (52.54 %)	19 (26.39 %)	
IV	2 (1.32 %)	0	2 (2.78 %)	
Tumor				0.048
T_1_	39 (25.83 %)	15 (25.42 %)	26 (36.11 %)	
T_2_	90 (59.60 %)	26 (44.07 %)	38 (52.78 %)	
T_3_	14 (9.27 %)	17 (28.81 %)	8 (11.11 %)	
T_4_	8 (5.30 %)	1 (1.70 %)	0	
Lymph node				0.041
N_0_	75 (49.67 %)	23 (38.98 %)	34 (47.22 %)	
N_1_	37 (24.50 %)	6 (10.17 %)	20 (27.78 %)	
N_2_	18 (11.92 %)	13 (22.03 %)	5 (6.94 %)	
N_3_	21 (13.91 %)	17 (28.82 %)	13 (18.06 %)	
Metastasis (at diagnosis)				0.467
M_0_	148 (98.01 %)	59 (100 %)	70 (97.22 %)	
M_1_	3 (1.99 %)	0	2 (2.78 %)	
Stage (at diagnosis)				0.007
Early	104 (68.87 %)	28 (47.46 %)	51 (70.83 %)	
Advanced	47 (31.13 %)	31 (52.54 %)	21 (29.17 %)	
Hormone receptor				
ER positive	108 (71.52 %)	37 (62.71 %)	38 (52.78 %)	0.022
ER negative	43 (28.48 %)	22 (37.29 %)	34 (47.22 %)	
PR positive	84 (55.63 %)	33 (55.93 %)	32 (44.44 %)	0.255
PR negative	67 (44.37 %)	26 (44.07 %)	40 (55.56 %)	
HER2				
Positive	41 (27.15 %)	17 (28.81 %)	23 (31.94 %)	0.333
Negative	96 (63.58 %)	32 (54.24 %)	48 (66.67 %)	
Borderline	14 (9.27 %)	10 (16.95 %)	1 (1.39 %)	
Recurrence				<0.001
No	134 (88.74 %)	48 (81.36 %)	59 (81.94 %)	
Yes	17 (11.26 %)	11 (18.64 %)	13 (18.06 %)	

### Risk analysis for BC patients with secondary diabetes and IFG

**Fig. 2:**
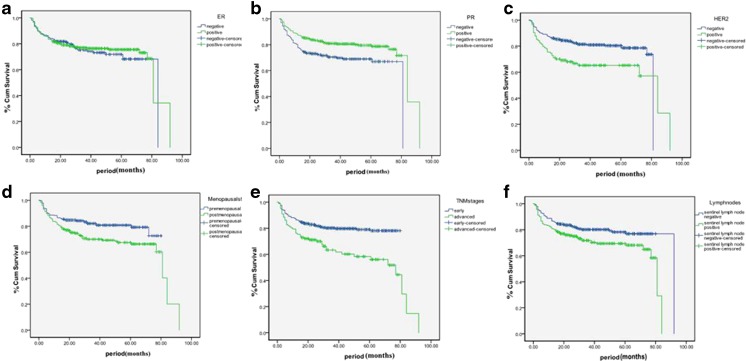
The risk rate for secondary diabetes and IFG in breast cancer patients with ER-positive group and ER-negative group. **a** The risk rate for secondary diabetes and IFG in breast cancer patients with PR-positive group and PR-negative group. **b** The risk rate for secondary diabetes and IFG in breast cancer patients with HER2 positive group and HER2 negative group. **c** The risk rate for secondary diabetes and IFG in breast cancer patients with premenopausal group and postmenopausal group. **d** The risk rate for secondary diabetes and IFG in breast cancer patients with sentinel lymph node metastasis group and without sentinel lymph node metastasis group. **e** The risk rate for secondary diabetes and IFG in breast cancer patients with early stage group and advanced stage group

A total of 59 patients developed secondary diabetes during a median follow-up of 41 months. Meanwhile, 72 patients developed secondary IFG, a status of prediabetes, which was also associated with an increased risk of cancer [17]. All factors for each group were included in the Kaplan–Meier analysis, and there were significant differences in clinicopathological features including menopausal status (*P* = 0.003); pathological stages (*P* < 0.001); lymph node status (*P* = 0.009); and expression of PR, ER, and HER2 (*P* = 0.004, 0.599, and 0.001, respectively) (Fig. 2). Table 3 shows descriptives for the conditional probability of developing diabetes over time in patients with BC treated with comprehensive therapy after adjustment for age and BMI. The risk of developing secondary diabetes for subjects with advanced BC (hazard ratio (HR) = 1.623; 95 % confidence interval (CI) 1.128–2.335 (*P* < 0.01)) was higher than for those with early BC. Although the risk of developing secondary diabetes for patients with negative PR expression (HR = 0.530; 95 % CI 0.372–0.755 (*P* < 0.001)) was higher than for those with positive PR expression, the risk of developing secondary diabetes for patients with negative ER expression was slightly higher than for those with positive ER expression (HR = 0.806; 95 % CI 0.566–1.146 (*P* = 0.230)), which was not statistically significant. Furthermore, the risk of developing secondary diabetes for the patients with positive HER2 expression (HR = 1.822; 95 % CI 1.230–2.700 (*P* < 0.01)) was higher than for those with negative HER2 expression. The risk of developing secondary diabetes for subjects with lymph node metastasis (HR = 1.595; 95 % CI 1.128–2.258 (*P* < 0.01)) was higher than for those without lymph node metastasis. Menopausal status did not significantly modulate systemic therapy–diabetes risk association. Therefore, pathological stages, lymph node metastasis status, hormone receptor expression, and HER2 expression were independent risk factors for developing secondary diabetes for the BC patients after systemic therapy.

**Table 3:** Median onset time and 95 % confidence intervals for secondary diabetes and IFG after systemic therapy for breast cancer patients

	Median onset time (months, 95 % CI)	*P* values	*P* values (adjusted for age and BMI)
TNM stages			
Early	65.667 (62.867, 68.468)	0.000	0.009
Advanced	54.880 (47.667, 62.093)		
Menopausal status			
Premenopausal	66.111 (62.678, 69.543)	0.003	0.111
Postmenopausal	61.736 (56.208,67.265)		
ER			
Negative	64.153 (59.576, 68.730)	0.599	0.230
Positive	67.551 (61.036, 74.066)		
PR			
Negative	58.921 (54.263, 63.578)	0.004	0.000
Positive	71.312 (65.877, 76.746)		
HER2			
Negative	67.085 (64.212, 69.958)	0.001	0.003
Positive	59.677 (51.666, 67.688)		
Lymph nodes			
Negative	74.828 (70.826, 78.830)	0.009	0.008
Positive	60.264 (75.368, 86.632)		

### Follow-up and clinical outcome

Complete follow-up information was collected from all the patients in this study. The median follow-up was 41 months (16–66). Of the 59 patients with preexisting diabetes, 94.7 % (54/59) received anti-diabetes therapy and 8.5 % (5/59) recovered to normal FPG levels after using diet and exercise. Two patients discontinued chemotherapy due to severe diabetes-related adverse effects. Overall, 189 patients died during the observation period; 7.95 % (12/151) of those with preexisting diabetes, 13.56 % (8/59) of those with secondary diabetes, 14.1 % (162/1152) of those with normal FPG, and 9.72 % (7/72) of those with secondary IFG (*P* = 0.070). The recurrence percentage of BC patients is significantly higher in the secondary diabetes group (*P* < 0.05, Table 2). Three patients with secondary diabetes developed bilateral primary breast cancer, compared with two patients in the preexisting diabetes group. One secondary diabetes patient developed squamous cell lung cancer, and one preexisting diabetes patient developed ovarian cancer.

## Discussion

BC and type 2 diabetes share similar etiologies, including hyperinsulinemia, hyperglycemia, and chronic inflammation [18]. Previous studies showed that both insulin resistance and diabetes positively affect cancer incidence [19–22]. However, the effect of BC and the use of systemic therapies for BC on the development of diabetes remains unclear [13]. In this study, we retrospectively investigated the potential association between systemic therapy and the incidence of type 2 diabetes in Chinese females with BC. A total of 1434 BC patients were followed up for a mean period of 41 months. Data revealed that the risk of developing diabetes after systemic therapy for the BC patients was higher than that for the normal control group (1.4 %), suggesting the potential association between systemic therapy and secondary diabetes in Chinese patients with BC. This increased incidence of secondary diabetes was associated with advanced BC stage at initial diagnosis.

A recent investigation revealed that the prevalences of diabetes and prediabetes in Chinese adults, 20 years of age or older, were 9.7 % (10.6 % among men and 8.8 % among women) and 15.5 % (16.1 % among men and 14.9 % among women), respectively [23]. A study in Canada reported that 16.5 % (1011/6107) of women with breast cancer aged 55–79 were diagnosed with diabetes at diagnosis, which is higher than the prevalence of 10.5 % (151/1434) in our study [7]. This suggests that the level of economic development and different lifestyles and diets may affect the prevalence of diabetes in different populations. However, patients’ age and the tendency for obesity were both considered to be risk factors for diabetes [7].

Recently, Ji et al. investigated the incidence of diabetes and prediabetes in 119 Chinese female adult BC patients after systemic treatment and found prevalences of 21.8 and 43.7 %, respectively [24]. Although these observations support our finding that patients with BC after systemic therapy tended to develop secondary diabetes, the increased incidence of diabetes was higher than in the current study. There are several possible reasons for this discrepancy. Firstly, they measured 2 h plasma glucose levels through OGTT, which were increased in 80.0 % of patients with normal FPG levels. Secondly, they investigated only 119 BC patients, which was not a large cohort of breast cancer survivors. Finally, the mean follow-up duration was only 18 months in the study.

Similar to previous studies [25], our results indicated that breast cancer patients with preexisting diabetes tended to have a high percentage of older, menopausal, and obese patients. In addition, breast cancer patients with diabetes were more likely to have larger tumor size, later pathological stages, a higher rate of lymph node metastasis, and a higher rate of ER-negative expression, which was also consistent with the findings of Hou et al. [25]. In our study, later pathological stages and more lymph node metastasis in the diabetic breast cancer group indicate that hyperinsulinemia promotes tumor cell proliferation and metastasis to some extent, which may be associated with interaction between insulin and its receptor, which may active the PI3K and Ras–MAPK pathways. Therefore, this study supports the hypothesis that diabetes is a high-risk factor affecting the growth of breast tumors and axillary lymph node metastasis. Therefore, systemic treatments for the breast cancer patients with diabetes have to be tailored to the individual, and strict control of blood sugar level is absolutely important.

Furthermore, our study suggests that secondary diabetes was associated with later pathological stages, more lymph node metastasis, and higher incidence of distance recurrence. The risk of developing abnormal plasma glucose levels after systemic therapy was significantly higher in patients with advanced stages than in patients with early stages. Patients with secondary diabetes were associated with a significantly higher risk of distance metastasis than patients with preexisting diabetes. One possible explanation for this is that hyperglycemia, hyperinsulinemia, and inflammation occur in newly diagnosed diabetes, which can result in tumor cell proliferation and metastasis [26–30]. However, hyperglycemia and hyperinsulinemia may exist rarely in patients with preexisting diabetes because blood glucose levels are usually controlled well using oral hypoglycemic agents or insulin. Nevertheless, because we did not have detailed information regarding insulin and glucose levels or the dose of oral hypoglycemic agents or insulin, further experiments are needed to be carried out to understand the underlying mechanism.

Very limited literature is currently available regarding the risk of secondary diabetes among breast cancer survivors. A study [12] provides evidence for an increased incidence of diabetes among postmenopausal breast cancer survivors, at age 55 or older, especially for patients who received adjuvant chemotherapy. Our results indicated that breast cancer patients with later pathological stages, more lymph node metastasis, negative hormone receptor expression, and positive HER2 expression tended to have a higher percentage of risk to develop secondary diabetes and IFG. Actually, the BC patients with the above clinical pathological characteristics, which are associated with poor prognosis, may receive more cycles of standard chemotherapy in the current study, which may contribute to the increased incidence of developing secondary type 2 diabetes. This study supports the hypothesis that BC patients after adjuvant chemotherapy experienced a higher rate of diabetes, at least in part, which is consistent with results of the previous study [12]. This conclusion is supported by a number of recent observations. First, Feng et al. reported that 5-FU-based chemotherapy might affect beta cell function by causing acute beta cell damage at the early phase and chronic damage at the later phase [31]. Second, other types of chemotherapy agents such as vinblastine and taxanes, which target microtubules and microfilaments, may inhibit the release of insulin and then increase blood sugar levels [32]. In addition, glucocorticoids are used commonly to reduce allergic or gastrointestinal adverse reactions during chemotherapy, which may cause temporary hyperglycemia [33–37]. Furthermore, the weight gain caused by high-energy diet and estrogen suppression during chemotherapy may further promote diabetes [38–40]. Finally, the impaired hepatic and renal functions caused by chemotherapy were suggested to be risk factors for the development of diabetes [41]. Nevertheless, the lack of a control group of BC patients without chemotherapy in our study means that the precise mechanism for our findings is unclear, and so further studies using an additional control cohort are needed to explore this relationship.

Nonetheless, our study has some limitations. It is important to note that the current study may be affected by the limited sample size (1434 cases) from three centers in one area. An additional national multicenter trial is needed to achieve a definitive conclusion regarding the effect of adjuvant therapy for BC patients on the incidence of secondary diabetes. It was reported that secondary diabetes was associated with significantly increased mortality [4]; however, we did not observe this association. This may be due to the different follow-up times, as the relative risk of diabetes for BC patients may increase over time [14]. In addition, we also did not have information regarding the use of prescription drugs, the doses of chemotherapy agents (such as vinblastine, taxanes, or glucocorticoids), or the dose of endocrine therapies such as tamoxifen, which was associated with an increased risk of diabetes among older females [42–45].

In summary, this large population-based study shows an increase in the incidence of diabetes among breast cancer survivors after systemic therapy, especially the patients with later pathological stages, more lymph node metastasis, negative hormone receptor expression, and positive HER2 expression. It is possible that chemotherapy is a factor leading to the increased risk of diabetes among the patients receiving systemic treatment. The reasons for this relationship are uncertain, and further research is necessary. In the meantime, greater diabetes screening and prevention strategies among breast cancer patients after systemic treatment are needed in China.
